# LYMPHADENO-DUODENAL FISTULA IN TUBERCULOSIS

**DOI:** 10.1590/0102-672020190004e1570

**Published:** 2021-05-14

**Authors:** Bipadabhanjan MALLICK, Dibya L PRAHARAJ, Preetam NATH, Sarat C PANIGRAHI, Anil C ANAND

**Affiliations:** 1 Department of Gastroenterology and Hepatology, Kalinga Institute of Medical Science, Bhubaneswar, India

**Keywords:** Lymphadeno-duodenal fistula, Tuberculosis, Endoscopy, Fístula duodenal, Tuberculose, Endoscopia

## INTRODUCTION

Tuberculosis is still one of the leading cause of morbidity and mortality in developing countries^1^. Patients with human immunodeficiency virus (HIV) tuberculosis infection can infect unusual sites and can have unusual presentations. Though tuberculosis can involve any part of the gastrointestinal tract, duodenal involvement is uncommon^2^. Tuberculous lymphadenitis leading to a fistulous communication with duodenum is sparsely reported in literature. We here report a case of tubercular lymphadenitis eroding into the duodenal wall causing lymphadeno-duodenal fistula. 

## CASE REPORT

**FIGURE 1:**
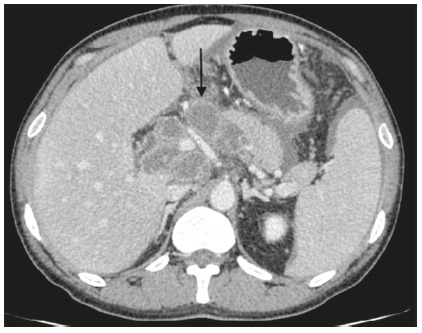
Abdominal tomography showing large necrotic periduodenal and peripancreatic lymph nodes (black arrow)

**FIGURE 2:**
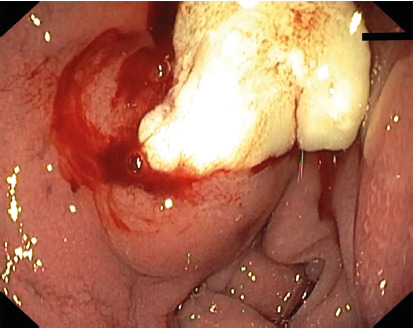
Upper gastrointestinal endoscopy showing lymphadeno-duodenal fistula

A 42-year-old man presented with fever, abdominal pain and distention for one month duration. He was also having history of anorexia and weight loss of 5 kg over one month. Systemic examination was negative for lymph nodes but was significant for ascites. He was tested positive for HIV infection with CD4 count of 70 cells/µl. Laboratory parameters showed hemoglobin of 11.3 gm/dl, total leukocyte count of 8.4 x 10^9^/l, normal renal and liver function. Contrast enhanced computed tomography of abdomen showed presence of ascites with multiple necrotic mesenteric, peripancreatic, periduodenal lymph nodes and omental thickening ( Figure 1). Ascitic fluid analysis showed serum ascites albumin gradient of 0.8 gm/dl, cytology negative for malignant cells, adenosine deaminase (ADA) of 23 u/l and cartridge based nucleic acid amplification test (CB-NAAT) was negative for *mycobacterium tuberculosis*. Upper gastrointestinal endoscopy done five days after abdominal tomography showed a bulge with thick whitish discharge from an opening in the duodenal bulb (Figure 2). CB-NAAT of the aspirated whitish material was positive for *mycobacterium tuberculosis*. He was started on anti-tuberculosis therapy and subsequently on antiretroviral therapy resulting in improvement of all symptoms. Repeated upper gastrointestinal endoscopy after six weeks showed complete resolution of duodenal fistula. 

## DISCUSSION

The periduodenal lymph node groups are commonly involved in abdominal tuberculous lymphadenitis^1^ ^,^ ^2^. The lymphadeno-duodenal fistula formation is a rare entity encountered in clinical practice despite the common involvement of periduodenal lymph nodes in tuberculosis^3^ ^,^ ^4^.There is formation of caseous necrosis in tuberculous lymphadenitis and these tuberculous lymph nodes can erode the adjacent duodenum resulting in a fistulous tract between the lymph node and duodenum^5^. Contrast enhanced tomography helps in identifying the cause of lymphadenopathy as well as the fistulous tract between the lymph node and duodenum^3^ ^,^ ^4^. In our case, abdominal tomography did not reveal any fistulous communication between the lymph node and duodenum but upper gastrointestinal endoscopy identified the fistula suggesting a freshly developed fistulous communication. This fistula can be either a presenting feature or can develop after starting anti-tuberculous therapy^3^ ^,^ ^4^. Management of the fistula require no additional specific therapy other than anti-tuberculous therapy^3^ ^-^ ^5^. 

To conclude, this case signifies that tuberculosis can involve unusual site or have unusual presentation in HIV infection. Lymphadeno-duodenal fistula is a rare presentation of tuberculous lymphadenitis. Fluid from the fistula site can be used for diagnosis of tuberculosis.
